# Silent myelin-weighted magnetic resonance imaging

**DOI:** 10.12688/wellcomeopenres.15845.2

**Published:** 2020-08-13

**Authors:** Tobias C. Wood, Nikou L. Damestani, Andrew J. Lawrence, Emil Ljungberg, Gareth J. Barker, Ana Beatriz Solana, Florian Wiesinger, Steven C.R. Williams

**Affiliations:** 1 Department of Neuroimaging, King's College London, London, UK; 2 Department of Psychological Medicine, King's College London, London, UK; 3 ASL Europe, GE Healthcare, Munich, Germany

**Keywords:** ihMT, Silent MRI, Myelin, ZTE, RUFIS

## Abstract

**Background:** Inhomogeneous Magnetization Transfer (ihMT) is an emerging, uniquely myelin-specific magnetic resonance imaging (MRI) contrast. Current ihMT acquisitions utilise fast Gradient Echo sequences which are among the most acoustically noisy MRI sequences, reducing patient comfort during acquisition. We sought to address this by modifying a near silent MRI sequence to include ihMT contrast.

**Methods: **A Magnetization Transfer preparation module was incorporated into a radial Zero Echo-Time sequence. Repeatability of the ihMT ratio and inverse ihMT ratio were assessed in a cohort of healthy subjects. We also investigated how head orientation affects ihMT across subjects, as a previous study in a single subject suggests this as a potential confound.

**Results:** We demonstrated that ihMT ratios comparable to existing, acoustically loud, implementations could be obtained with the silent sequence. We observed a small but significant effect of head orientation on inverse ihMTR.

**Conclusions: **Silent ihMT imaging is a comparable alternative to conventional, noisy, alternatives. For all future ihMT studies we recommend careful positioning of the subject within the scanner.

## Introduction

Myelin is a critical part of a healthy nervous system and hence visualising it *in vivo* is of great use to clinicians. Fortuitously, myelin displays multiple physical properties that give rise to contrast in Magnetic Resonance (MR) images. Tissue containing myelinated axons has lower longitudinal and transverse relaxation times ^1, 2^, lower susceptibility ^3^, increased Magnetization Transfer (MT) effects ^4^, and reduced diffusion ^5^, compared to non-myelinated tissue. However, while all of these MR parameters are sensitive to myelination, they are not specific, as other biological processes demonstrate the same effects ^6^. An additional MR-relevant property of myelin is that it is semi-crystalline in nature, being formed of closely packed proteins and lipids ^7^. This regular structure can maintain dipolar order, and recent work has shown that this can be exploited to produce inhomogeneous Magnetization Transfer (ihMT) contrast ^8– 10^. Although other substances and tissues such as muscle can exhibit ihMT ^11, 12^, it is possible to tune the acquisition parameters specifically to the properties of myelin ^13^. This, along with the fact that there are no other candidate substances that can exhibit ihMT within the central nervous system, suggests that ihMT has the potential to produce genuinely myelin-specific contrast ^8^.

Previous work has shown that within a single subject the ihMT effect exhibits a dependence on the orientation of axons to the magnetic field ^10, 14, 15^. This is attributable to the preferential alignment of myelin sheaths with the axons, leading to a non-uniform distribution of orientations of the lipids and proteins in the sheath ^16, 17^. As the orientation of WM to the magnetic field will also depend on the orientation of the head, this positioning of the patient within the scanner may influence ihMT metrics. To our knowledge, this effect of bulk orientation across subjects has not been directly investigated.

Recent ihMT imaging methods utilise fast gradient-echo sequences to acquire full-brain images in a reasonable time frame ^18, 19^. Such sequences are among the loudest MR sequences as they utilise a short Repetition Time (TR) and require rapid switching of high amplitude field gradients ^20^. Acoustic noise is a leading cause of discomfort for subjects during an MR examination ^21, 22^, and is of particular concern in paediatric and fetal MRI ^23– 26^.

It is possible to make 3D gradient echo acquisitions almost silent by swapping from the standard Cartesian to a radial zero echo-time (ZTE) acquisition scheme ^27– 29^, but due to the fixed (near zero) echo-time and RF amplifier limits it can be difficult to achieve strong tissue contrasts in such sequences ^30, 31^. In the current work we incorporate an MT preparation module into a radial ZTE sequence without compromising the acoustic noise level and show that myelin-weighted contrast can be achieved at the expense of only a small increase in scan time. The primary aim was to measure the repeatability of semi-quantitative ihMT and inverse ihMT ratios ^14^. As a secondary aim we hence investigated the effect of head orientation across subjects on the ihMT effect.

## Methods

### MR sequence

**Figure 1.**
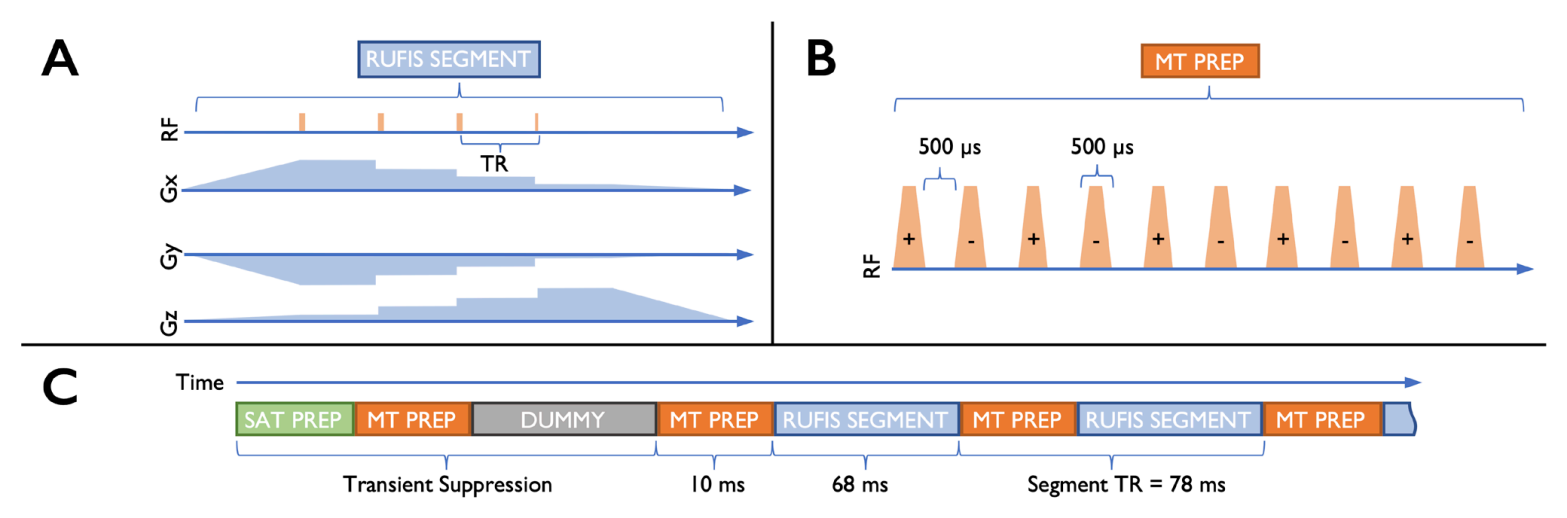
**A** - Sequence diagram for a RUFIS segment. The gradients are first ramped to a constant amplitude, and short hard RF pulses are used to avoid slab profile effects from the constant gradient magnitude. For clarity, only four spokes are illustrated in the segment, our acquisition had 32. **B** - The MT preparation block consists of multiple saturation pulses. To generate the ihMT effect the sign of the saturation frequency is alternated, for standard MT preparation all pulses would have the same sign. **C** - The overall sequence consists of RUFIS segments separated by MT preparation blocks. At the start of each image a saturation module nulls all signal, and dummy segments where no data is acquired are played until the steady-state magnetization is reached.

The Rotating Ultra-Fast Imaging Sequence (RUFIS) was originally introduced to image flowing liquids ^27^. It is essentially a gradient echo sequence, where each TR consists of a single RF pulse followed by a readout. The principal difference, illustrated in Figure 1, is that the readout gradient is held constant during the TR, including during the excitation pulse, and hence each readout consists of a ‘spoke’ that starts in the center of k-space and moves towards one edge. This is in contrast to a standard Cartesian sequence where the imaging gradients acquire a line from one side of k-space to the other.

The innovations introduced in order to be robust to flow effects have numerous serendipitous effects, chiefly massively reduced acoustic noise levels compared to conventional MR imaging schemes ^29^. As shown in Figure 1A, the imaging gradients have constant magnitude and only their orientation is varied during the sequence in order to appropriately sample k-space. At the end of each TR the gradient direction is changed by a small step on each gradient channel. As there are no rapid or large gradient changes, which are the major source of acoustic noise, RUFIS acquisitions are extremely quiet.

Because Radio Frequency (RF) excitation occurs in the presence of the imaging gradients in RUFIS, high bandwidth excitation pulses are required to mitigate slab profile effects. In practice, very short hard pulses (on the order of 10µs) are required, which limits the range of available flip-angles to a few degrees due to RF amplifier and transmit coil limitations ^30^. This restricts a naïve RUFIS implementation to Proton-Density (PD) weighting ^31^. To circumvent this limitation, the sequence can be segmented, with preparation pulses played in between segments; this approach has previously been utilised for T2 ^32^ and diffusion prepared RUFIS imaging ^33^.

MT is a common technique for generating contrast in tissues that have high fractions of non-aqueous hydrogen protons ^4, 34^. Such tissues include White Matter (WM) in the brain, and so MT has seen wide application in WM diseases ^35, 36^. The most common acquisition method is a Cartesian gradient echo sequence with an off-resonance saturation pulse added to every TR ^37^. It is not feasible to play a saturation pulse in every TR within RUFIS due to the constant presence of the imaging gradient. If a saturation pulse was played while this gradient was present, the effective frequency offset of the pulse would differ across the field of view. Instead, we added a train of saturation pulses, shown in Figure 1B as a preparation module before each segment.

The approach of prepared segmented MT imaging has already been demonstrated with a Cartesian readout ^38^, and can be tuned to produce an increased sensitivity to myelin by varying the width and spacing of the saturation pulses ^12^. Cartesian readouts can choose their *k*-space view order to preferentially weight the center of *k−*space to the MT effect. Because the center of *k*-space is sampled in every repetition in RUFIS, such view re-ordering is not possible. T1 recovery during a segment is hence a significant issue in RUFIS, as it will dilute any weighting from a preparation module. We therefore used a short segment length to minimise T1 recovery. This results in playing the preparation pulses more frequently, which lengthens scan time. However, MT preparation requires very little dead time compared to either T1 or T2 preparation, and so the overall increase in scan time is minimal.

To minimise any contamination from transient signals at the start of scanning or when switching between different MT preparation schemes, we adopted two complementary measures. The first was the addition of a saturation module played once at the start of each volume. This consisted of a single adiabatic 90 degree pulse and spoiler to effectively null all longitudinal magnetization. Following this module, 48 dummy segments were played, where the no data was acquired, to allow the signal in brain parenchyma to approach a steady-state. The 48 dummy segments lasted for 3.3 seconds, which is longer than the approximate T1 of parenchyma (around 1s) at 3T. The full sequence schematic is shown in Figure 1C.

### Imaging study

We recruited 6 male and 6 female subjects (age range 25 to 54 years) through local advertisement within our research establishment (King’s College London). Subjects gave written informed consent in accordance with ethics approved by the King’s College London REC (approval number 04/Q0706/72), and standard MRI exclusion criteria were applied. Each subject had two imaging sessions, spaced approximately a week apart, in a 3 Tesla scanner using a 12-channel head coil (Discovery MR750, GE Healthcare). Each session consisted of two ihMT scans, for a total of four ihMT scans per subject. In addition to the ihMT data, to provide an anatomical reference, in the first imaging session a standard T1-weighted image was acquired using the ADNI-GO protocol ^39^. No special instructions were given to subjects as to their head positions in order to acquire a representative sample of orientations with respect to the main magnetic field.

The ihMT scans consisted of five images (see below). All volumes were acquired with the following parameters: 22cm field of view, 1.5mm isotropic voxel size, readout bandwidth ±25kHz, TR 1.764ms, spokes-per-segment 32. We used a 2° hard pulse for excitation. This was lengthened from the manufacturer default of 8µs to 24µs to lower the B1 amplitude and hence minimise any saturation of the bound pool from the excitation pulses. A train of 10 Fermi saturation pulses was played between each segment with pulse-width of 500µs and a 500µs gap in between. The pulses had a root-mean-square B1 of 8.75µT and an offset frequency of 7kHz. This corresponds to a root-mean-square B1 of 6.2µT over the course of the preparation module. The total RUFIS segment time and preparation time (including ramps and switching time) were 68.6ms and 10.8ms respectively. We did not add an explicit spoiling gradient after the pulse train, instead relying on the initial gradient ramp of the acquisition segment to spoil spuriously generated transverse magnetization from the MT pulses.

To isolate the ihMT contrast images acquired under both single-sided and dual-sided irradiation are required. The five volumes were acquired with the following scheme saturation scheme: +/-, -/+, none, +, -, where + or - refers to the sign of the saturation offset frequency. We refer to the volumes with only positive or negative saturation frequency as MT-weighted and those with dual-sided saturation as enhanced MT (eMT) weighted. Scan time per ihMT volume was 65 seconds, and 5 minutes 41 seconds for all five volumes. The acoustic noise was measured with an MR-compatible microphone (Casella CEL-63X, IDEAL Industries) located in the scanner bore.

Previous work has shown that ihMT ratio (ihMTR) is sensitive to confounds from B1 (RF transmit inhomogeneity) and T1-weighting, but this can be potentially mitigated through the use of an inverse ihMTR ^14^, and such inverse metrics have also been used to compensate for T1 effects in Chemical Exchange Saturation Transfer experiments ^40^. To calculate this we additionally acquired a volume with T1-weighting. Because it is difficult to achieve high flip-angles with the short block pulses in RUFIS ^30^, we opted to replace the ihMT preparation train with a single on-resonance 10ms 25° pulse. The on-resonance preparation pulse generates a large amount of unwanted transverse magnetization compared to the off-resonance MT pulses, and so we added a 10 cycles-per-voxel spoiler gradient which increased the preparation module time to 11.3ms. The spoiler gradient ramp time was lengthened to reduce acoustic noise to the level of the rest of the RUFIS sequence.

The RUFIS images were reconstructed using the manufacturer’s proprietary Orchestra toolbox (GE Healthcare). The reconstruction consisted of nearest-neighbour gridding on a twice-oversampled grid ^41^, *k*-space center filling ^42, 43^, and Total Generalised Variation (TGV) regularization ^33, 44^. The TGV regularisation parameter was set to λ = 0.01 with a maximum of 64 iterations.

### Analysis

We first motion corrected the MT-weighted images with mcFLIRT ^45^. The MTR, eMTR, ihMTR, inverse ihMTR and MT-asymmetry were calculated using an open-source C++ program added to QUIT ^46^. MT-asymmetry is a measure of whether the absorption rate differs for positive or negative irradiation frequencies ^47^. The parameters were defined to match ^14^:

$$\text{MTR}=1-\frac{S_{+}+S_{-}}{2S_{0}}\;(1)$$

$$\text{eMTR}=1-\frac{S_{+/-}+S_{-/+}}{2S_{0}}\;(2)$$

$${\text{MT}}_{\text{asym}}=\frac{S_{+}-S_{-}}{S_{0}}\;(3)$$

ihMTR=2(eMTR−MTR)(4)

$${\text{ihMTR}}_{\text{inv}}=2S_{T1}\left(\frac{1}{S_{+/-}}+\frac{1}{S_{-/+}}-\frac{1}{S_{+}}-\frac{1}{S_{-}}\right)\;(5)$$

where *S*_+_, *S*_−_, *S*_+/−_ and *S*_−/+_ refer to the signal from the saturation schemes defined above, and *S*_*T*1_ is the signal from the T1-prepared image.

We calculated an affine transform from the eMTR image to each subject’s standard T1-weighted image using ANTs ^48^. The eMTR image was selected because the contrast is broadly similar to the T1-weighted. We then constructed a study template from all subject’s T1-weighted images, and non-linearly registered the resulting image to the MNI atlas ^49, 50^. Analysis then proceeded in two complementary directions: first, for illustrative purposes, we resampled each subject’s MT metrics in MNI space, and second, for a quantitative region of interest (ROI) analysis we resampled the JHU WM atlas into the native space of each scanning session. To minimise the number of resampling operations all transforms between the MNI space and the MT-weighted native space were concatenated before application. Ten bilateral white matter tract ROIs were selected ^51^.

Mean average value within the ROI was calculated for each ROI at each of the 4 scans (2 repeats at 2 sessions) for MTR, eMTR, ihMTR and inverse ihMTR. Intra-class correlation coefficients (ICC) were calculated over the 4 measurements using the regularised mixed-effects method of 52. Specifically, two-way random ICC(2,1) values and 95% confidence intervals (bootstrap percentile method; 1000 resamples) were extracted from a random effects model with random effects of subject and scan. Regularisation of variance components was achieved via a weakly informative gamma prior (shape parameter 2, rate parameter 0.5) ^52^. Calculations were performed in R version 3.6.2 using the *blme* package version 1.0.4 ^53^. ICC values were classified as poor if they were less than 0.5, moderate if between 0.5 and 0.75, good between 0.75 and 0.9, and excellent above 0.9 ^54^.

Finally, we investigated how head orientation affects ihMTR. As a proxy for how each subject’s head was aligned with the main magnetic field, we calculated the angle between the Z-direction (head-foot axis) of the MT scan space and the MNI atlas. This was found by first concatenating the three affine transforms (MT- to T1-weighted, T1-weighted to study template, and study template to atlas), applying the concatenated transform to the vector *Z* = (0, 0, 1) and then calculating the dot product between the result and *Z*.

We only examined the effect of orientation on ihMTRinv, as this is expected to be robust against B1- and T1-effects ^14^, which may also have a spatial or angular dependence ^55^. To probe the impact of head orientation in the presence of potential between-participant differences, we calculated linear mixed effects models using *blme* as above. These models included mean ihMTRinv values from all 10 ROIs, main effects and interactions of head angle & ROI and both a random and fixed effect for each participant, with subject allowed to interact with ROI. Of prime interest was the average linear effect of head angle within-subject and whether this varied over the ROIs. Statistical significance was tested with ANOVA adjusted by the Kenwood-Roger procedure, with p<0.05 considered significant.

## Results

### Images

**Figure 2.**
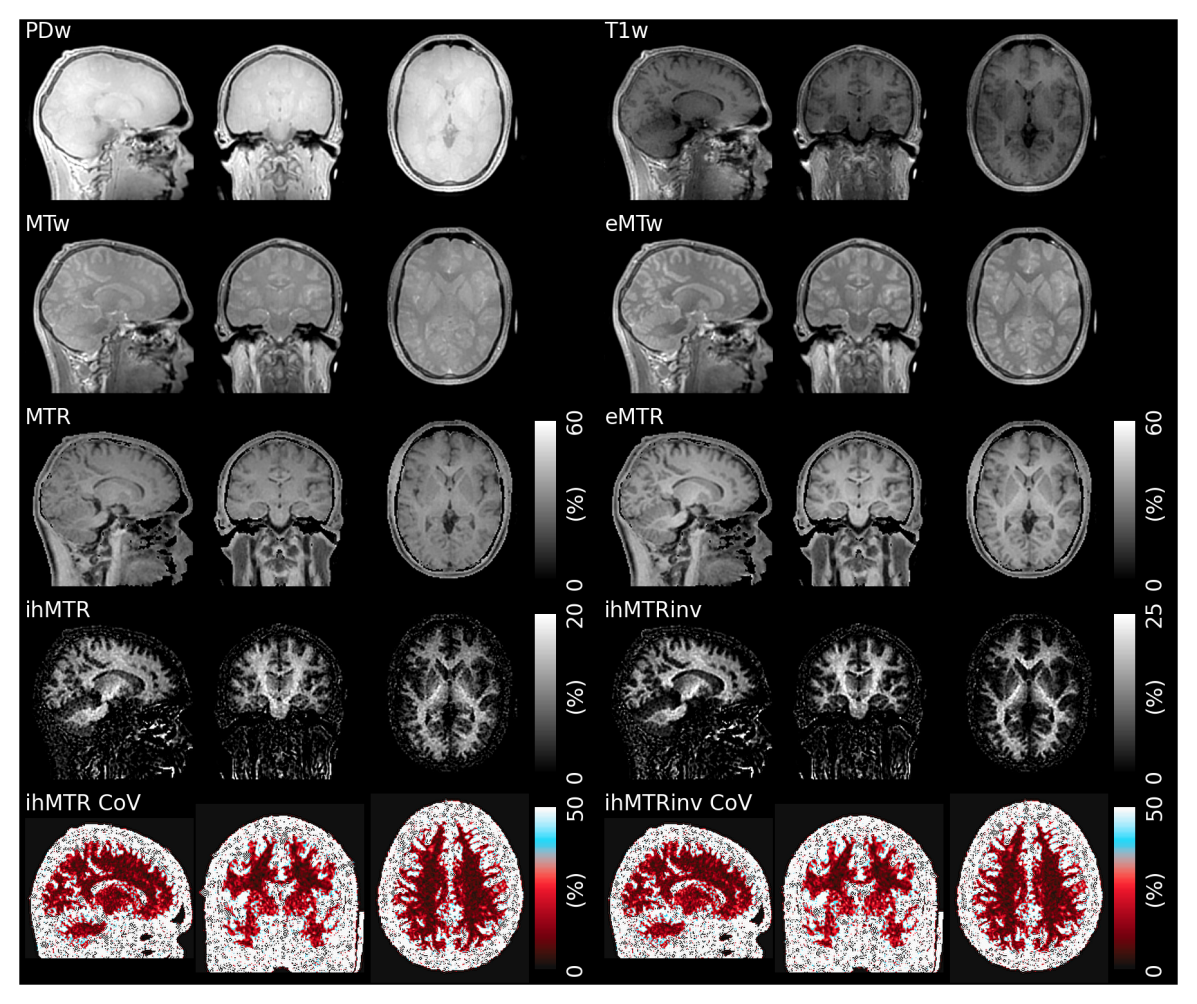
Example raw weighted images (PD, T1, MT and eMT), ratio images (MTR, eMTR, ihMTR and ihMTRinv) and within-subject CoV for one subject.

Figure 2 shows the acquired raw images and calculated MT ratios from a single subject. The acoustic noise was measured as 72 dB, compared to a 69 dB background level, which is similar to our previous work where comparable Cartesian sequences were approximately 30 dB louder ^30^.

The inverse ihMTR shows a subtly improved contrast between white and grey matter compared to ihMTR, particularly in the cerebellum and putamen (note the different color scale).

The eMT-weighted image in particular demonstrates good grey matter (GM)/WM contrast, while the MTR image exhibits little GM/WM contrast. The ihMTR, which is the difference between eMTR and MTR (with a scaling factor of two), hence also shows very good GM/WM contrast. It is very close to zero outside the brain, in contrast to both MTR and eMTR which are high in tissues outside the brain. The inverse ihMTR exhibits improved GM/WM contrast compared to ihMTR in the deep GM structures, such as the putamen, the cerebellum and reduced B1 inhomogeneity effects throughout WM.

**Figure 3.**
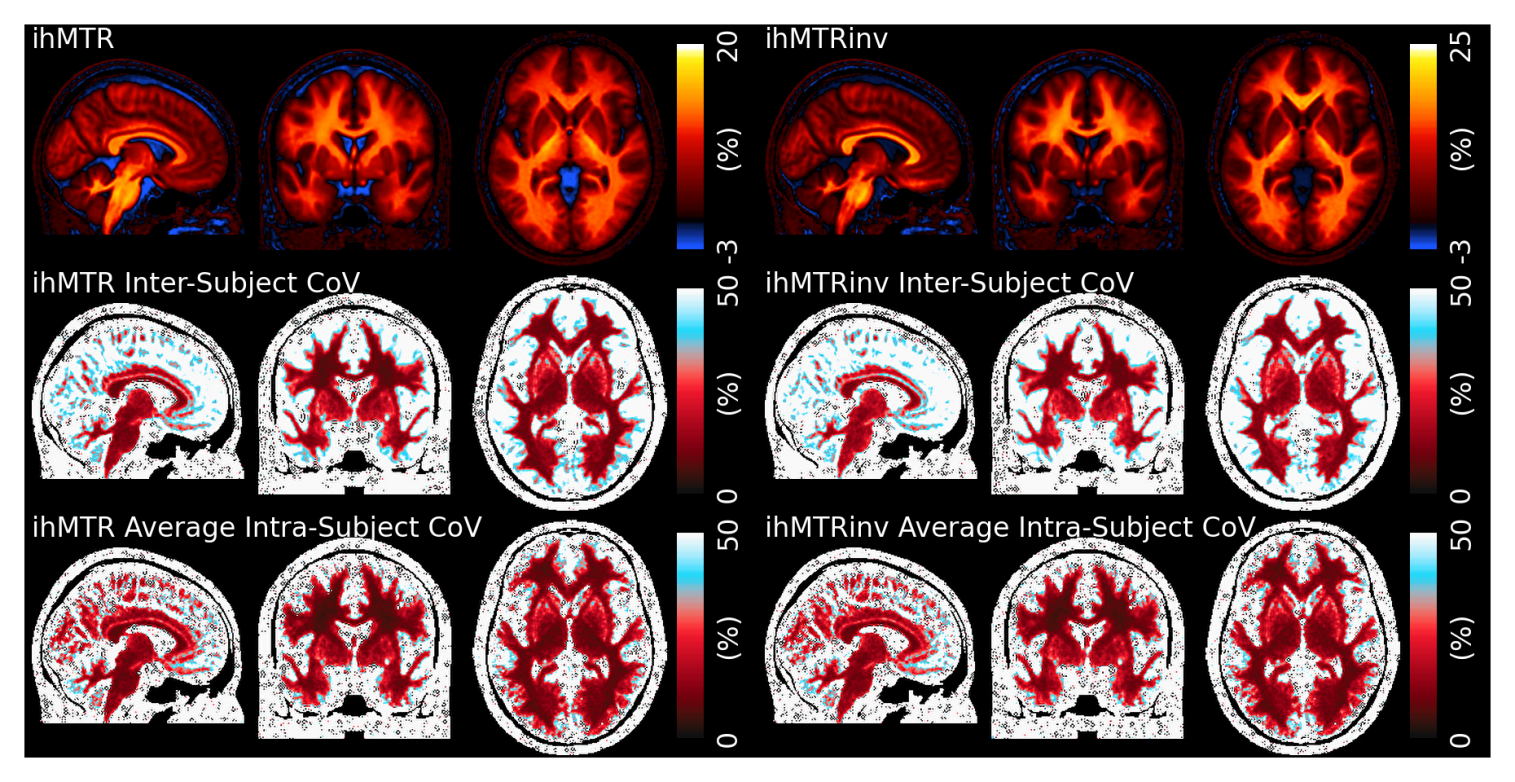
Top - ihMTR and ihMTRinv values averaged across all scans in MNI space. An asymmetric color scale has been used to highlight the small negative of ihMTR in cerebral spinal fluid. Heightened values can be observed in WM tracts oritented parallel to B0. Middle – Between-subject CoV calculated across all scans and subjects. Bottom – Average within-subject CoV. The within-subject CoV is lower than the between-subject in the cortex.

Figure 3 shows the mean, between-subject and average within-subject Coefficient of Variation (CoV) for ihMTR and inverse ihMTR in MNI space, while Table 1 summarises the average ihMTR and inverse ihMTR for the ROIs we examined. The ihMTR and inverse ihMTR values were, respectively, about 12% and 15% in WM, with values in tracts oriented parallel to the main magnetic field slightly higher as expected ^15^. Values were lower in GM, and we observed a small negative ihMTR in cerebral spinal fluid (CSF) and the eyeballs.

**Table 1.** The mean and standard deviation of ihMTR and inverse ihMTR in the 10 selected ROIs, calculated across all subjects.

ROI	ihMTR Mean (%)	ihMTR SD (%)	Inverse ihMTR Mean (%)	Inverse ihMTR SD (%)
**Genu of CC**	10.81	0.16	15.20	0.38
**Body of CC**	11.79	0.17	14.70	0.44
**Splenium of CC**	12.52	0.18	15.78	0.52
**Corticospinal** **tract**	15.18	0.08	18.52	0.58
**Cerebral** **peduncle**	13.89	0.17	15.14	0.48
**Internal Capsule**	12.67	0.22	15.47	0.53
**Corona Radiata**	12.67	0.16	16.54	0.40
**Thalamic** **Radiation**	12.17	0.19	15.67	0.48
**Cingl. Cingulate** **Gyrus**	10.82	0.15	13.30	0.40
**Cingl.** **Hippocampus**	10.49	0.41	10.49	0.76

The between-subject CoV was approximately 10% in WM, but approaches 50% in GM and reached over 100% in CSF. The average within-subject CoV was lower, at around 8% in WM and 30% in GM. The average value of inverse ihMTR is higher, at approximately 20%. The contrast in parenchyma is broadly similar to ihMTR, but there are subtle differences in deep GM, frontal WM and the brain stem and values in CSF are close to zero instead of negative. Both the between-subject and average within-subject CoV in WM is slightly higher than for ihMTR.

**Figure 4.**
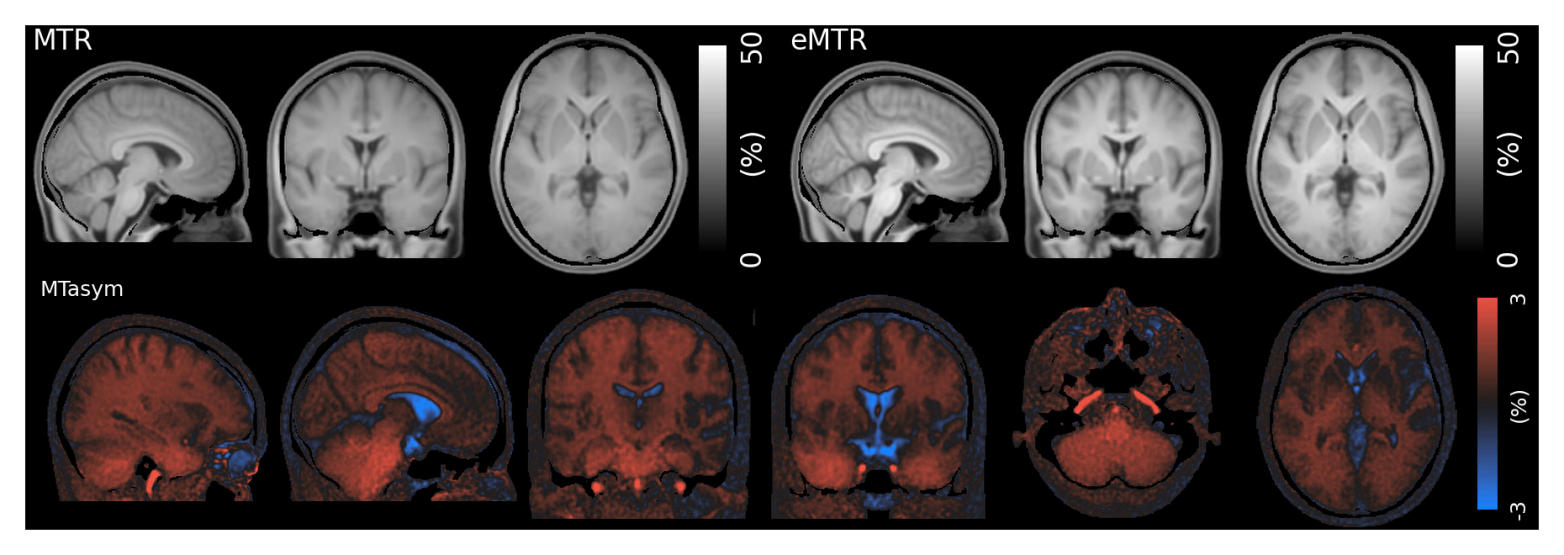
Mean MTR, eMTR and MT-Asymmetry in atlas space. Elevated levels of MT asymmetry can be observed in the ascending arteries and cerebellum.

Figure 4 shows the mean MTR, eMTR and MT-asymmetry. The MTR image shows only limited contrast between WM and GM despite the high levels of saturation power, while the eMTR image shows the expected improved contrast. In contrast to ihMTR, non-zero MTR and eMTR can be observed in tissues outside the brain. We found a small consistently positive value of MT asymmetry in cerebral WM, which was increased in cerebellar WM and the major ascending arteries. MT asymmetry was negative in CSF and the eyeballs.

### Reliability

**Figure 5.**
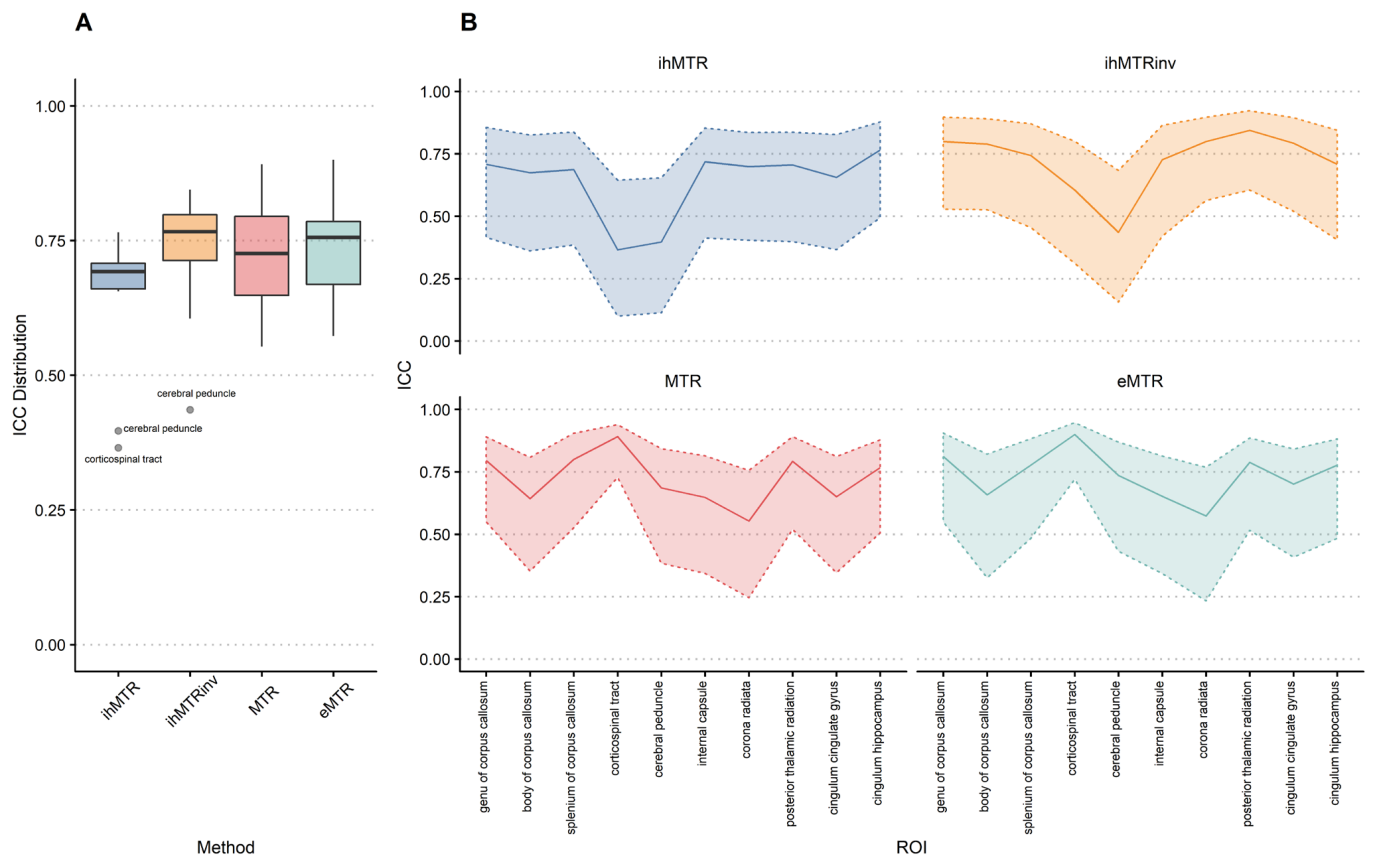
**A** - Intra-class correlation coefficient (ICC) distribution (Tukey Boxplot). **B** - ICC (solid line) and 95% bootstrap confidence intervals (dashed lines and shaded area), displaying a profile over the regions of interest (ROIs).

**Figure 6.**
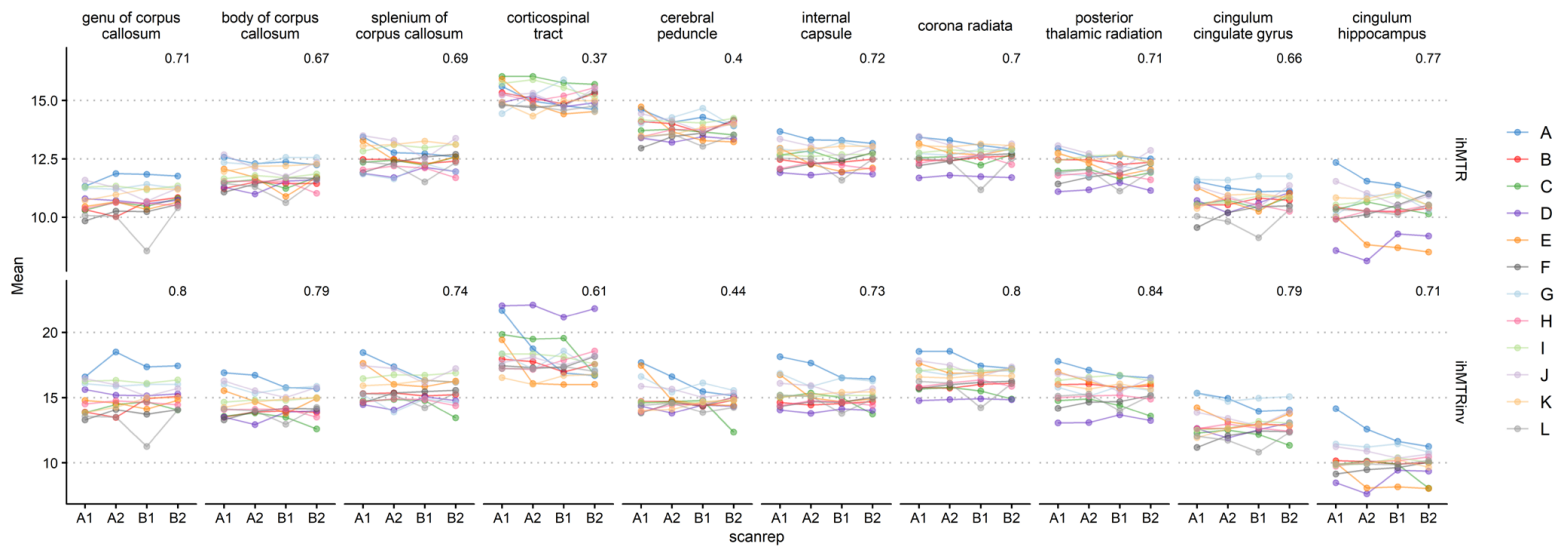
Subject-level variability in ihMTR and inverse ihMTR for the 10 atlas regions of interest.

Figure 5 shows the obtained ICC values in the atlas ROIs. ICC values were moderate or good for all measures, except for the cerebral peduncles in both ihMTR and inverse ihMTR and corticospinal tract in ihMTR only, where the ICC values were poor. ICC values were slightly higher for inverse ihMTR compared to ihMTR. The cerebral peduncle and corticospinal tract ROIs commonly gave results atypical of the remaining ROIs. Figure 6 shows the mean values of ihMTR and inverse ihMTR for all ROIs across all four scans. Most ROIs show good reliability and repeatability ^56^, but there are several obvious outliers, for instance subject D in the corticospinal tract for inverse ihMTR, and subjects D and E for ihMTR in the cingulum hippocampus.

### Head orientation

**Figure 7.**
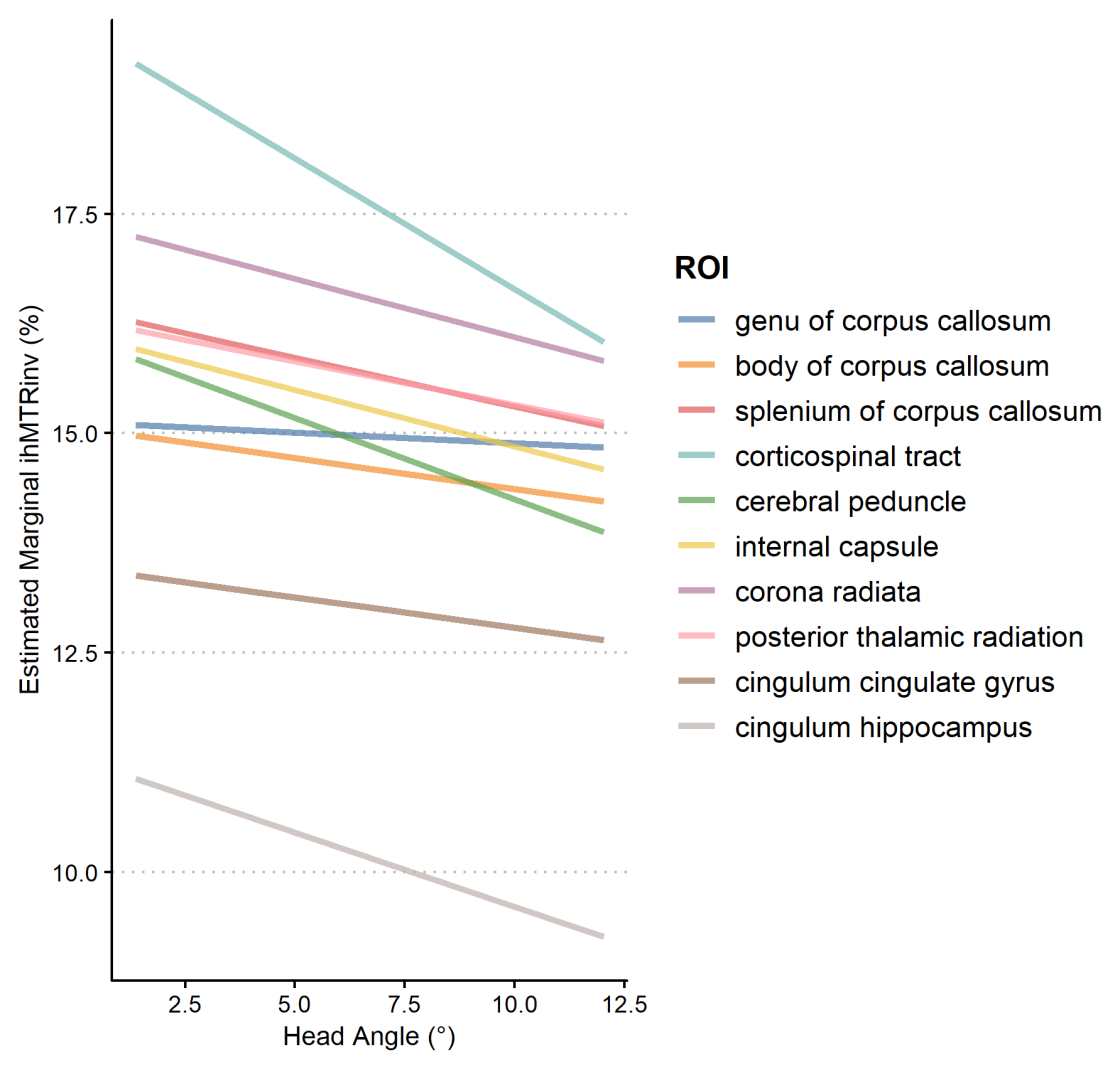
Estimated effects of participant head angle× region of interest (ROI) for inverse ihMTR. As the angle of the head increases, the value of inverse ihMTR tends to decrease.

The observed median head orientation angle was 7° (lower quartile 3.2°, upper quartile 10.3°). Subject D showed particularly elevated values around 25° (excluding subject D the maximum observed angle was 12°). Closer examination revealed that subject D had a fairly small head and in both sessions was scanned with their head tilted back within the coil (data not shown). Our analysis revealed a significant main effect of head angle ( *F* = 27.7, *p* = 2.50 *×* 10 ^−7^) on ihMTRinv, such that with increased rotation angle inverse ihMTR values were lower, illustrated in Figure 7. There was no significant interaction between angle and ROI ( *F* = 1.47, *p* = 0.15) indicating effects were relatively homogeneous over the ROIs. Because subject D (angle = 25°) could be interpreted as an outlier, we repeated the analysis with subject D excluded and results were comparable (main effect of angle: *F* = 37.24, *p* = 3.00 *×* 10 ^−9^; angle *×*ROI interaction: *F* = 1.32, *p* = 0.23).

## Discussion

We have demonstrated full-brain 3D myelin-weighted ihMT images acquired with a silent and fast imaging sequence. The MT preparation module increases scan-time by a minimal amount and does not compromise the silent nature of RUFIS acquisition. ihMT has shown potential for assessing myelination in multiple sclerosis ^57, 58^, and the use of a silent sequence will extend this potential to noise intolerant patient cohorts, for instance non-sedated infants.

The single-sided saturation MT-weighted and MTR images showed fairly flat contrast between WM and GM. This is to be expected, as the 7 kHz frequency offset chosen is optimal for the ihMT effect, whereas a smaller offset, for example 2 kHz, would likely generate larger MT contrast. Although the eMTR image exhibits good WM/GM contrast, it also shows significant signal in other tissues outside the brain, such as muscle, cartilage and blood.

Combining the eMT and MT-weighted images into an ihMT ratio increases the specificity of the sequence to myelin, as evidenced by an ihMTR close to zero outside the brain. Our ihMTR values, at around 12% in WM, are similar to previous literature using a comparable preparation module with a Cartesian readout ^13, 38^, but lower than recent papers using a low-duty cycle preparation module ^13, 18^. Our protocol was adapted from that presented in 38, which had comparable levels of power deposition during the preparation module, and the principal difference is our acquisition module acquires a larger number (32) of center-out readout spokes with a low flip-angle instead of a small number of Cartesian readouts with a higher flip-angle.

To mitigate against T1 recovery during the readout segment, which repeatedly samples the center of k-space, we minimised the number of acquired spokes to 32. The resulting segment time of less than 70 ms is much shorter than typical T1 times in parenchyma (approximately 1 s at 3T). Increasing the number of spokes per segment would lead to a reduction in scan-time, at a cost of increased T1 recovery and potentially reduced ihMTR. 

A particular drawback of radial ZTE sequences compared to Cartesian is constrained SNR. We observed some residual T1-weighted contrast in the PD-weighted reference image. Reducing the excitation flip-angle below 2° to further reduce the T1-weighting would incur a linear reduction in SNR (from the small flip-angle approximation sin *α* ≈ *α*), which would likely yield unacceptable image quality. We used Total Generalized Variation regularization in our reconstruction to primarily to improve image quality, whereas for Cartesian sequences such methods are generally used with parallel imaging to speed up acquisitions ^18, 38, 44^. Although non-cartesian parallel imaging methods exist ^59^, to our knowledge none has been specifically tailored to 3D radial acquisitions. Despite this limitation, we acquired 1.5 mm isotropic maps in 6 minutes, which is competitive with a recent cartesian ihMT acquisition with an MP-RAGE type readout which acquired 2.4 mm isotropic maps in the same time ^18^.

We observed a small negative ihMTR in CSF and the vitreous humour of the eyeball. The likely cause of this is a small, unwanted, difference in direct saturation effects between our single-sided and dual-sided irradiation preparation modules. Changing the shape of the preparation pulses to one with better controlled sidebands, for example Hann or Gaussian, would likely remove this effect ^38^. Use of the inverse ihMTR appeared to mitigate T1 and B1+ effects and led to improved contrast between WM and GM, and more consistent contrast within WM, for instance the internal capsule and genu of the corpus callosum have different ihMTR values but similar inverse ihMTR values. To fully determine whether the inverse ihMTR reduced B1+ contributions would require the acquisition of additional B1+ maps, which was beyond the scope of the current work.

We observed a large MT asymmetry in the carotid arteries and an elevated value in the cerebellum compared to the cerebrum. Blood is known to exhibit an MT effect, due to a high concentration of protein ^60– 62^, and this is also known to be asymmetric ^63^. PET and Dynamic Contrast Enhanced MRI measurements indicate that the cerebellum has increased vascularity and cerebral blood volume compared to the cerebrum ^64, 65^. Hence this result appears to be consistent with previous literature.

The CoV maps in Figure 3 showed high values in cortical GM, indicating poor reliability of ihMTR metrics in the cortex. This is partly to be expected due to the very small absolute values of ihMTR in both GM. However, the average within-subject CoV was lower than the between-subject CoV, indicating that partial volume effects and registration quality affected the between-subject figure. These issues are not unique to ihMTR but affect all quantitative MRI measures ^66, 67^.

The repeatability of both the ihMTR and inverse ihMTR, as defined by the ICC scores, fell on the boundary of the moderate (0.5-0.75) and good (0.75-0.9) categories with the exception of the cerebral peduncles and corticospinal tract which had notably worse scores. The ICC scores of inverse ihMTR were slightly superior to ihMTR. Our ICC values are lower than a repeatability study of a steady-state Cartesian ihMT study ^51^. However, the values are not directly comparable as that study used a 2.4×2.4×3.2 mm voxel size compared to our 1.5 mm isotropic voxel size, with a similar overall scan time. Our lower ICC values can hence at least be partly attributed to the smaller voxel size and correspondingly lower SNR.

As shown in Figure 6 most subjects had consistent measures across all four scans but others, notably subject A, showed high variability across sessions. This variation can at least in part be attributed to the orientation of the head with the main magnetic field. We found that the observed ihMTR decreased in all ROIs as the observed rotation angle of the head increased. Our method for quantifying the rotation angle is imperfect, as it cannot distinguish positive and negative rotations and the choice of the MNI template as “zero” is arbitrary. We also did not control for the average angle of each ROI, variations in tract orientation within an ROI, the effect of hemisphere, or the effect of inter-volume motion during the ihMT scan. Despite these limitations, we showed a small but highly statistically significant effect of angle on inverse ihMTR, and so conclude that head orientation is a potential confound in ihMT studies. Recent work has incorporated prospective motion correction into a cartesian ihMT sequence ^18^, and such approaches could be of benefit in this radial implementation to minimise both inter- and intra-volume motion artefacts in problematic patient cohorts (e.g. infants).

## Conclusion

We have demonstrated that MT-weighting can generate significant additional GM/WM contrast in silent ZTE images with minimal extension of scan time. We have shown that the derived semi-quantitative MT ratios have good repeatability, and that the inverse ihMTR has advantages over the ihMTR. However, the ihMT effect depends on the orientation of the subject’s head within the bore, and hence we recommend that careful attention is paid to participant’s positioning in future work.

## Data availability

### Underlying data

Figshare: Silent Myelin-Weighted MR Imaging, https://doi.org/10.6084/m9.figshare.12090645 ^68^.

This project contains the following data:

- Atlas images in MNI space format
- ROI summary statistics in Comma Separated Value format

Data are available under the terms of the Creative Commons Attribution 4.0 International license (CC-BY 4.0).
