# Supplementary material for: Silent myelin-weighted magnetic resonance imaging
Source: Wellcome Open Res. 2020 Aug 13;5:74. Originally published 2020 Apr 21. [Version 2] doi: 10.12688/wellcomeopenres.15845.2 (PMC7431975; doi:10.12688/wellcomeopenres.15845.2)
Supplement: Peer review history

# Peer review: Reviewer response for version 2

**DOI:** 10.21956/wellcomeopenres.17734.r39968

**Published:** 2020-10-16

**Authors:** Gunther Helms

**Affiliations:** 1 Medical Radiation Physics, Department of Clinical Sciences Lund, Lund University, Lund, Sweden

A module of 10 off-resonance Fermi pulses was integrated into a silent 3D radial ZTE sequence (RUFIS) to derive different metrics for magnetization transfer (MT) and inhomogeneous MT (ihMT) of the brain at 3T. It is demonstrated that MT impose additional GM/WM contrast onto the proton density-weighted ZTE images. From these, metrics of inhomogeneous MT (ihMT) are derived, which have been shown to correlate to myelin. Obviously, the presented methodology is especially suited to study myelination in children.  

 The sequence implementation is thoroughly motivated. A careful evaluation of 12 healthy adults was conducted to define sequence-specific semi-quantitative values for MT and ihMT.  Repeatability and susceptibility to angulation is described both for individuals and on a group level. The presented documentation may be regarded exemplary for implementation of ihMT into the context of an imaging sequence.

Is the rationale for developing the new method (or application) clearly explained?

Yes

Is the description of the method technically sound?

Yes

Are the conclusions about the method and its performance adequately supported by the findings presented in the article?

Yes

If any results are presented, are all the source data underlying the results available to ensure full reproducibility?

Yes

Are sufficient details provided to allow replication of the method development and its use by others?

Yes

Reviewer Expertise:

Quantitative MRI, especially MT.

I confirm that I have read this submission and believe that I have an appropriate level of expertise to confirm that it is of an acceptable scientific standard.

# Peer review: Reviewer response for version 2

**DOI:** 10.21956/wellcomeopenres.17734.r39965

**Published:** 2020-08-20

**Authors:** Richard Dortch

**Affiliations:** 1 Division of Neuroimaging Research, Barrow Neurological Institute, Phoenix, AZ, USA

The authors have addressed all of my critiques. I have no new comments.

Is the rationale for developing the new method (or application) clearly explained?

Partly

Is the description of the method technically sound?

Yes

Are the conclusions about the method and its performance adequately supported by the findings presented in the article?

Partly

If any results are presented, are all the source data underlying the results available to ensure full reproducibility?

Yes

Are sufficient details provided to allow replication of the method development and its use by others?

Yes

Reviewer Expertise:

Imaging science, quantitative MRI, myelin imaging.

I confirm that I have read this submission and believe that I have an appropriate level of expertise to confirm that it is of an acceptable scientific standard.

# Peer review: Reviewer response for version 2

**DOI:** 10.21956/wellcomeopenres.17734.r39967

**Published:** 2020-08-17

**Authors:** Douglas Dean

**Affiliations:** 1 Department of Pediatrics, University of Wisconsin-Madison, Madison, WI, USA; 2 Department of Medical Physics, University of Wisconsin-Madison, Madison, WI, USA; 3 Waisman Center, University of Wisconsin-Madison, Madison, WI, USA

The authors have addressed my previous comments and the manuscript is much improved. I have no further comments.

Is the rationale for developing the new method (or application) clearly explained?

Yes

Is the description of the method technically sound?

Partly

Are the conclusions about the method and its performance adequately supported by the findings presented in the article?

Yes

If any results are presented, are all the source data underlying the results available to ensure full reproducibility?

Yes

Are sufficient details provided to allow replication of the method development and its use by others?

Partly

Reviewer Expertise:

quantitative Magnetic Resonance imaging, white matter imaging.

I confirm that I have read this submission and believe that I have an appropriate level of expertise to confirm that it is of an acceptable scientific standard.

# Peer review: Reviewer response for version 1

**DOI:** 10.21956/wellcomeopenres.17381.r38686

**Published:** 2020-06-04

**Authors:** Gunther Helms

**Affiliations:** 1 Medical Radiation Physics, Department of Clinical Sciences Lund, Lund University, Lund, Sweden

An MT-module of 8 off-resonance Fermi pulses was integrated into a 3D silent zero TE radial scan (RUFIS) to derive different metrics for magnetization transfer (MT) and inhomogeneous MT (ihMT). Since is MT is tissue specific, it is demonstrated that MT-weighting can be used to generate additional GM/WM contrast in silent ZTE images, which per se show poor tissue contrast due to low flip angle.

 The paper is well motivated, carefully conducted, and presented clearly.

 The reviewer has three points of concern:

1. Since MT is not specific for myelin and the higher specificity of ihMT for myelin is not fully established yet, a more conservative descriptive title is recommended, e.g. Inhomogeneous MT (ihMT) in silent ZTE MRI.
2. Via the saturation of the bound pool/dipolar reservoir, the imposed saturation by MT will depend on spatial B _1_^+^ inhomogenieties as acknowledged in the paper. Since the 3D images/maps are presented only by sagittal views, it is impossible for the reader to appreciate the residual spatial inhomogeity of the metrics. Transversal images and maps of (ih)MT metrics should also be presented. Since the paper specifically address the influence of orientation relative to B0, it would be good to compare this effect in size to the influence of the local deviation from the nominal B _1_^+^ (varying between 80% and 120% across the brain at 3T). Variation in B _1_^+^ can be mimicked by manipulating the transmit gain. 
3. To mitigate these spatial effects, the authors calculate an inverse ihMT metric using a reference ZTE image, where T1-weighting has been imposed in the preparation module. However, this metric is not identical to the one in Ref 20, which is based on a simplified signal equation that approximates a sequence where single MT pulses and SPGR readouts are interleaved (Helms *et al*. 2008 ^1^). To complicate matters, Ref 20 did not show theoretically that their metric actually reduces B _1_^+^. Qualitative arguments become clear only after lengthy reconstruction, as the inverse MTR (reference of the same flip angle) is proportional to the MTsat. Since the framework of Helms *et al*. is not applicable to interleaved ZTE , the authors should mention the pragmatic nature of borrowing this approach. NB that comparing non-selective partial saturation by 1-cos (B _1_^+^*25°) = approx 0.95*B _1_^+2^ to tissue-type-dependent repetitive absorption (roughly proportional to B _1_^+^^_2_^) seems reasonable well motivated to the reviewer, but will like obscure T1 relaxation effects even more than MTR-like metrics using S0.

Minor points:

1. Intro: For the uninitiated reader, the “technical limitations” precluding strong ZTE contrast may be mentioned already in the introduction.
2. Methods: Please provide the measurement time required for one 3D ZTE volume (I calculated about 1:45 mins) and the total of 5 weightings.
3. Methods: Ref 20 used -/+ 5kHz offset. Please motivate the use of -/+ 7kHz. (My guess is shorter MT pulses.)
4. Methods: It would be helpful for the uninitiated reader to motivate the definition (sign) of MT _asym _Hua, Jones, Blakely *et al*. 2007 ^2^ is the earliest report of asymmetric MT in brain.
5. The CV map in second right, lower row panels in Figures 3 and 4 do not seem to match the maps in the top row. CV maps 3 and 4 do, so please replace 5.
6. The dependence on surrogate head angle in Fig. 8 is largest in cortico-spinal tract (parallel to B0) and smallest in corpus collosum ROIs (always perpendicular to B0). It would be helpful to state this finding in the flow text.

Is the rationale for developing the new method (or application) clearly explained?

Yes

Is the description of the method technically sound?

Yes

Are the conclusions about the method and its performance adequately supported by the findings presented in the article?

Yes

If any results are presented, are all the source data underlying the results available to ensure full reproducibility?

Yes

Are sufficient details provided to allow replication of the method development and its use by others?

Yes

Reviewer Expertise:

Quantitative MRI, especially MT.

I confirm that I have read this submission and believe that I have an appropriate level of expertise to confirm that it is of an acceptable scientific standard, however I have significant reservations, as outlined above.

## References

[1] : High-resolution maps of magnetization transfer with inherent correction for RF inhomogeneity and T1 relaxation obtained from 3D FLASH MRI. *Magn Reson Med*.2008;60(6) : 10.1002/mrm.21732 1396-407 10.1002/mrm.21732 19025906

[2] : Quantitative description of the asymmetry in magnetization transfer effects around the water resonance in the human brain. *Magn Reson Med*.2007;58(4) : 10.1002/mrm.21387 786-93 10.1002/mrm.21387 17899597PMC3707117

# Response

**Published:** 2020-08-13

**Authors:** Tobias C. Wood

**Affiliations:** King's College London, UK

We thank the reviewer for their time and insight. There were in total five reviewers, with many helpful suggestions, and hence there have been many edits to the paper. Responses to this particular review follow below.

1. We agree that MT is not specific for myelin, and while there is increasing evidence that ihMT can be made specific to myelin (e.g. Duhamel et al 2019), we opted for caution in the title “Silent myelin- *weighted* magnetic resonance imaging” (emphasis added) because myelin is likely to be the dominant cause of contrast. We think that this is justified in comparison to T1-weighted and T2-weighted imaging, where T1 and T2 are the dominant but not sole contrast mechanisms. Hence we opt to keep the title unchanged.

2. We have changed the 3 sagittal views of ihMTR and ihMTRinv to sagittal, axial & coronal views. In our opinion, visually, these show good spatial homogeneity. In our opinion assessing the deviation due to B1+ would be best assessed with the acquisition of a separate B1+ map, which due to time constraints could not be included in this study.

3. The suggested reference has been added. Additional references describing an asymmetric MT effect in blood have been added and the discussion amended accordingly.

Minor Points

1. The fixed echo-time and RF power limitations have been explicitly added to the introduction.

2. The volume measurement time is present in the text “Scan time per volume was 65 seconds”, we have added the total acquisition time for 5 volumes.

3. A range of offsets for the ihMT pulses have been used in the literature. Reference 14, Mchinda et al 2018, indicated that an offset frequency higher than 5 kHz produced a larger ihMT effect, in fact finally opting for 8 kHz. In early sequence testing 7 kHz was found to produce a sufficient ihMT effect.

4. The suggested reference has been added. In addition we have updated the discussion about the asymmetry effect in light of finding an additional reference on the topic (Zhou et al 2005).

5. We do not completely understand this comment. In figure 5, the bottom row is the MT-asymmetry, not a CoV map.

6. The lowest effect is present in the cingulum hippocampus, not the corpus callosum. The colour scheme for these two ROIs was quite similar, it has been amended.

# Peer review: Reviewer response for version 1

**DOI:** 10.21956/wellcomeopenres.17381.r38685

**Published:** 2020-05-29

**Authors:** Douglas Dean

**Affiliations:** 1 Department of Pediatrics, University of Wisconsin-Madison, Madison, WI, USA; 2 Department of Medical Physics, University of Wisconsin-Madison, Madison, WI, USA; 3 Waisman Center, University of Wisconsin-Madison, Madison, WI, USA

Overview: This study describes the development of a novel, silent inhomogeneous magnetization transfer (ihMT) technique for imaging myelin in a clinically feasible time. Specifically, the authors evaluate the reproducibility of ihMT derived metrics from a cohort of healthy subjects. The authors additionally explored the influence of head orientation on ihMT measures, as recent study has suggested this to possibly affect these measures. A total of 4 scans were acquired from each participant (2 sessions, 2 ihMT scans per session) and the authors examined ihMT metrics across the whole brain as well as from regions of interests (ROIs) from an existing brain atlas. Intra-class correlations were used to assess reliability. The authors demonstrate comparable and good reliability of ihMT metrics from the novel, silent sequence, while coefficent of variation of ihMT and inverse ihMT demonstrate high consistency of the measures, particularly in white matter. An association with head orientation was also observed. Overall, the manuscript is well written, the study is well designed, and appropriate methodologies were used. The results demonstrate the proposed silent ihMT method could provide a reliable alternative to conventional ihMT strategies. However, I do have several points of concern that I believe could be addressed through a minor revision: 

 Comments:

1. Prior to computing the ihMT metrics, source images are motion corrected. Given the finding that the ihMT metrics are dependent on head orientation, I’m curious about how intra-scan motion (i.e. orientation changes between source images) may affect the measurements as well? Was there motion between source images during the initial motion correction step? This may be particularly relevant for the populations of interest (e.g. infants/young children/elderly) where motion may be more likely.
2. A study specific template was constructed from the (standard) T1-weighted images, with this study template subsequently registered to the MNI atlas. Which methods/software were used to create the study specific template? Was the study specific template registered to MNI using linear or nonlinear registration methods? 
3. For resampling the MT metrics to the MNI space, was this performed in a single interpolation step? Or were transformations applied in multiple steps?
4. The coefficient of variations of Figures 3 and 4 in white matter look good, however, the values in gray matter, particularly the cortex, seem very high. The authors mention this is expected given small ihMTR in gray matter and that registration quality may also impact these values. This seems to suggest that gray matter ihMT values may be unreliable and that analyses with ihMT metrics should be restricted to white matter. This may be particularly relevant given recent interest in examining cortical microstructure measures. Also, were coefficient of variations calculated by combining all subject data together or were within subject coefficient of variations computed (from the 4 scans) and then averaged? If data were combined together, how do the within subject coefficient of variations look?
5. When reporting the ihMTR and inverse ihMTR values, it would be informative to provide a table of means and standard deviations either from broad tissue types (e.g. gray matter, white matter, whole brain) and/or from the white matter regions that were examined. This would allow a reader to see these values more readily and make them more accessible.
6. Minor Comment: What was the timing between scan sessions? It appears that measures and reliability were consistent within-session and across-sessions. Is this accurate?
7. Minor Comment: Page 5 – I’d consider a new paragraph with the sentence: “Finally, we investigated how head orientation affects ihMTR.”
8. Minor Comment: Figure 2: The red-yellow color scale of the calculated MT metrics makes it difficult to compare the contrast of these metrics with the weighted images, as described in the results. 
9. Minor Comment: For statistical analyses, was a p-value of p<0.05 considered statistically significant? Were corrections for multiple comparisons (with multiple ROIs being tested) taken into account?
10. Minor comment: Page 6, first paragraph: I believe “slight” should be “slightly”.

Is the rationale for developing the new method (or application) clearly explained?

Yes

Is the description of the method technically sound?

Partly

Are the conclusions about the method and its performance adequately supported by the findings presented in the article?

Yes

If any results are presented, are all the source data underlying the results available to ensure full reproducibility?

Yes

Are sufficient details provided to allow replication of the method development and its use by others?

Partly

Reviewer Expertise:

quantitative Magnetic Resonance imaging, white matter imaging.

I confirm that I have read this submission and believe that I have an appropriate level of expertise to confirm that it is of an acceptable scientific standard, however I have significant reservations, as outlined above.

# Response

**Published:** 2020-08-13

**Authors:** Tobias C. Wood

**Affiliations:** King's College London, UK

We thank the reviewer for their time and insight. There were in total five reviewers, with many helpful suggestions, and hence there have been many edits to the paper. Responses to this particular review follow below.

1. We did not attempt to quantify how intra- & inter-volume motion affected the ihMT measurements and have now noted this in the discussion section. The amount of inter-volume motion was minimal in this study, likely because the subjects were well behaved adults. Intra-volume motion in 3D radial images generally manifests as blurring, due to the repeated acquisition and implicit averaging of the center of k-space. We have added a sentence and reference to the discussion section on this topic. 

2. The study specific template was created using the antsMultivariateTemplateConstruction2 script, which is the method associated with the given citation. Non-linear registration was used to align this with the MNI template, this has now been noted in the text.

3. Transforms were concatenated before being applied in a single step. This has now been stated explicitly in the text.

4. Thank you for the suggestion to look at the average within-subject CoV. Figure 2 and the new figure 3 have been expanded to include this. The within-subject CoV is smaller, particularly in the cortex, than the between-subject CoV. This indicates that cortical registration failures are increasing the between-subject CoV in the cortex. The discussion has been amended in light of this.

5. A table of the average ihMTR and inverse ihMTR values in our selected ROIs has been added.

6. The spacing between sessions (approximately 1 week) has been added to the methods section.

7. The suggested change has been made.

8. In Figure 2 we now have used a greyscale colormap for all images except the CoV maps to aid comparability. We have kept the two-way colormap for figure 3 due to the negative CSF value issue.

9. There is only one statistical hypothesis in the paper – does ihMTRinv vary with head orientation? As this is a single F-test across all the acquired ROI data, it does not require multiple comparisons correction. p<0.05 was considered significant, and this has now been stated explicitly in the methods. The test was repeated twice, once with a potential outlier excluded, and the final p-values were several orders of magnitude below the significance threshold. The ICC values do not require MCC.

10. The typo has been corrected.

# Peer review: Reviewer response for version 1

**DOI:** 10.21956/wellcomeopenres.17381.r38619

**Published:** 2020-05-27

**Authors:** Olivier Girard, Lucas Soustelle

**Affiliations:** 1 CNRS, CRMBM UMR 7339, Aix-Marseille University, Marseille, France; 2 Aix-Marseille Univ, CNRS, CRMBM UMR 7339, Marseille, France; 3 SATT Sud-Est, Marseille, France

This paper describes an original implementation of the inhomogeneous magnetization transfer (ihMT) MRI technique within a Zero Echo Time (ZTE) pulse sequence allowing for strong reduction of acoustic noise during MRI acquisition. This allows generating myelin sensitive ihMT images with a silent MRI sequence to scan sensitive patient. The sequence is designed for neuroimaging and offers a whole brain coverage with a 1.5mm isotropic voxel size in a relatively short scan time of about 5 to 6 minutes. Whereas 1/ whole brain ihMT sequences based on cartesian gradient echo readout and 2/ ZTE imaging sequences, such as RUFIS used here, have been previously described in the literature, the combination of an ihMT module within a silent ZTE sequence is original and should make ihMT contrast available to patient population that are specifically sensitive to loud noise such as young children and elderly, which is important for brain maturation study for instance.

 The sequence is demonstrated on a small cohort of healthy volunteers (N=12) and the study includes a repeatability assessment, with and without volunteer repositioning. This allows assessing the robustness of the technique as well as the confounds associated with the head orientation within the scanner bore.

 The paper is well written and the presented data are rather convincing, however there are several concerns that need to be addressed in our opinion.

- General concerns:

1/ About the systematic error observed in CSF

 The negative ihMTR signal observed in CSF is likely a systematic error, which I believe is not related to MT effect in CSF. This raises concern about the sequence implementation which should be checked. First of all I doubt there is a highly significant MT effect in CSF (ref 46, does not seem appropriate to document this, but there are problems with the references in the current version of the paper), and I believe that “MT effects” observed in CSF are in major part due to direct saturation effects, especially when using routine clinical sequences for conventional MT experiments which use relatively low frequency offset to perform the saturation (which is not the case here). Besides, although it is correct that most previous ihMT studies have not focused on CSF, some have included CSF in the analysis (e.g. Girard et al.MRM 2017 ^1^), and the observed CSF ihMT signal was basically close to zero within measurement errors. Second, there is no theoretical reason why the ihMT signal would be negative; this would mean that the dual offset saturation would be less efficient than the single offset, which contradicts Provotorov theory.

 However, there is a plausible explanation to the negative signal observed in CSF related to differential direct saturation effects obtained with single and dual offset MT experiments. When using a train of pulses the frequency response of the RF saturation not only depends on the carrier frequency of the MT pulses and on the timings of the pulse train, but also on the phase of each pulse, which means that the on-resonance (or close to resonance) component of the MT excitation will not be identical for single and dual offset MT experiment, and also for negative and positive offset single MT experiment. Together with the specific B0 shimming conditions this could explain unexpected ihMT and MT asymmetry signal in CSF and blood.

 Direct saturation effects occurring with ihMT sequences may be mitigated with the use of a gradient spoiler after each burst of MT pulses, but in the proposed implementation the author do not use such a spoiler gradient and it is not clear whether readout gradients are sufficient to prevent direct saturation effects.

 In order to check this hypothesis, and also as a general check of the sequence implementation, we encourage the author to perform a very single in vitro experiment: reproduce their ihMT acquisition on a non-MT sensitive doped aqueous sample (ideally with a size comparable to the human head, and a T2 value close to the free pool in vivo T2) using the same parameters as for the in vivo experiment, and check the direct saturation effects of each individual MT experiment (negative, positive, and dual offset). Ideally, author could also add in the field of view a MT responsive sample (like agar gel) and ihMT responsive sample (like commercial hair conditioner that are made of lamellar liquid crystals). Basically, there should be no MT signal, nor MTA, nor ihMT signal for the aqueous sample.

 All this being said, it is agreed that inflow effects could also bias MT and ihMT metrics and that other causes may explain the observe systematic error, but we believe that performing the suggested in vitro experiment will help identifying the cause of the systematic error.

 2/ On the head orientation study

 The scope of the head orientation study is limited, and the conclusion seems somewhat overstated. First of all the topic is not introduced with enough details and the study design only allows to extract very limited information on that topic: the only message from the paper is that the head orientation may bias ihMT metric and that one should be careful when positioning the patient. Whereas we agree that the head orientation has an effect on the ihMT signal and that one should be careful with patient positioning, the authors’ conclusions are too subjective as they conclude that the effect is “strong” without providing any quantitative comparison with other source of errors. We invite the author to expand a little more the introductory part of the study, and to be more objective in their conclusion. The abstract should be rephrased accordingly as well.

 3/ Comparison with existing literature

 The comparison with ihMT metrics from previous literature is limited. The authors should expand the discussion on that topic. This is particularly important here since a new readout is proposed to probe the ihMT contrast. Although major differences in ihMTR are not expected with different readouts or weightings from our experience, providing that the sequence is run in “steady state” and that the RF power associated with readout pulses is low, some effects such as the k-space view ordering and associated point spread function may bias the measured signal. The author should discuss the specifics of the RUFIS readout (effects of radial UTE-like vs. standard cartesian GRE/SE signal acquisition) and how these affect the measured signal, ideally by providing simulations on the effect of the RUFIS readout module on ihMTR as functions of the segments composition (TR/number of shots/flip angle).

- Specific comments: 

Introduction: The introductory part of the angular dependency of ihMT should be expanded. The paper provides very limited explanations on the origin of this effect. This has been partly documented previously (Girard *et al*. (2017) ^2^) on the basis of the non isotropic lineshape of white matter introduced by Pampel et al. NIMG 2015 ^3^.

 Method/MR sequence: Author should provide more details about the “transient suppression” method that they used (fig. 1C), and provide argument or simple simulations so as to determine why “48 dummy segments” are sufficient to generate a steady-state.

 Method/Imaging Study: “ […] we found the gradient ramp was insufficient to remove residual transverse magnetization and so we added a 10 cycles-per-voxel spoiler gradient which increased the preparation module time to 11.3ms. The spoiler gradient ramp time was lengthened to reduce acoustic […]” Did the author consider adding the same spoiler gradient for the ihMT prep? (see general comment above)

 Method/analysis: “As a proxy for how each subject’s head was aligned with the main magnetic field, we calculated the angle between the Z-direction (head-foot axis) of the MT scan space and the MNI atlas.“ This is an interesting approximation. However, in my opinion the main issue is that it misses the assessment of individual tracts orientation, which is necessary to address the angular dependency of ihMT.

 Method/Analysis related to the head orientation study: Some part of this section should appear in introduction as they are not specific to the method: “Previous work has shown that the inhomogeneous MT effect partially depends on the orientation of myelin with the main magnetic field 10,20,21,as assessed by a voxel-wise comparison to diffusion data. To our knowledge, the effect of bulk orientation across subjects has not been directly investigated.”

 In addition, Ref 20 and 21 do indeed show orientation dependency of ihMT as evaluated from diffusion data ; however Ref 10, explains how the residual dipolar coupling vary with the orientation, but does not specifically addresses the angular dependency of ihMT.

 Method/analysis: “We only examined the effect of orientation on ihMTRinv, as this is expected to be robust against B1- and T1-effects 20, which may also have a spatial or angular dependence 43.” Unfortunately, the B1 sensitivity of the high flip angle reference inverse ihMT ratio (ihMTRinv) is still under investigation and may not be as robust as initially thought. In addition, the sequence may have different sensitivity to B1 depending on the sequence parameters (see Mchinda et al. MRM 2018 ^4^). In the presented study the ihMTRinv images from fig 2 show a strong hyperintense area located in the middle of the brain which looks somewhat colocalized with typical B1+ patterns observed @ 3T, as for the corresponding ihMTR images. This raises concern regarding the choice of ihMTRinv instead of ihMTR for the analysis. In addition, the residual dependency of ihMTRinv to B1 + shall be further discussed.

 Discussion: “The single-sided saturation MT-weighted and MTR images showed fairly flat contrast between WM and GM. We attribute this to T1 relaxation effects during the segment, and residual T1-weighted contrast in the PD-weighted reference image. […] Switching from single-sided to dual-sided saturation increases contrast between WM and GM …“

 It is surprising the authors do not mention dipolar order to explain the lack of contrast on the single-sided MT-weighted MTR images. Indeed, these effects act against saturation of the macromolecular pool, hence reducing MTR signal and contrast on single sided MT experiments. This is the key difference between single and dual sided MT experiments. Of interest, the MT experiments run in this study are not comparable to usual MT since relatively high frequency offsets and low duty cycle were used here. These condition favor dipolar order and hence are an important determinant of GM/WM contrast.

 Discussion: “… Our ihMTR values, at around 15% in WM, are broadly in line with previous literature using a Cartesian sequence “ Previous literature has shown that ihMT depends on many sequence parameters. This should be contextualized here. Also it is hard to compare with previous literature without providing the rms B1 calculated over the sequence repetition time.

- Minor points: 

Abstract:

 “Silent ihMT imaging is a comparable alternative to conventional, noisy, alternatives.” Comparable should be removed.

 Introduction:

 “This, along with the fact that there are no other candidate substances that can exhibit ihMT within the nervous system, suggests that ihMT has the potential to produce genuinely myelin-specific contrast”. Please specify “central nervous system”.

 p3: reference to citation 11 about ihMT is likely inappropriate.

 Methods:

 MR sequence: “… high bandwidth excitation pulses are required to avoid introducing a slab profile” and in caption of Fig 1A. Please use “mitigate” instead of “avoid”.

 MR sequence: “… increased ihMT effect in myelin ” Please use WM instead of myelin, this is a shortcut toward considering that ihMT is a purely myelin-specific effect.

 Imaging study: typo: plural “6 females, 6 males”.

 Imaging study, first paragraph: we suggest to make explicit here the reason why the ihMT scans were repeated twice (repeatability) and repeated again in another session (head orientation study).

 Imaging study: “We used a 2°, 24μs (low B1 amplitude) hard pulse for excitation to minimize any saturation of the bound pool from the excitation pulses”: the sentence sounds misleading since shorter RF pulses lead to stronger B1RMS for identical flip angle, please rephrase.

 Imaging study: was the choice of Fermi pulse motivated by any specific criteria? Since there are free parameters in the Fermi window function, the authors should provide more information on the pulse shape, such as bandwidth and power integral (or B1peak and B1rms is preferred).

 Imaging study: please provide the root-mean-square B1 of MT pulses calculated over the segment TR (ihMT prep + readout) to ease comparison of presented results with existing literature.

 Imaging study: we suggest the author describe first that ihMT rely on comparing single and dual frequency MT experiment, before describing the five volumes to be acquired. Currently the ihMT method description may be difficult to follow for non-expert ihMT users.

 Analysis: reference to citation 36 about the QUIT toolbox is likely inappropriate. Please check all references.

 Analysis: “Previous work has shown that the inhomogenous MT effect partially depends on the orientation of myelin with the main magnetic field”. typo: “inhomogeneous”.

 Results:

 Images: No acoustic noise measurement was reported for the T1w-RUFIS acquisition.

 p6 typo error: “The CoV in WM was below 10% in WM”. WM is repeated twice.

 “the very small absolute values of ihMTR in both GM and CSF, …”..It is misleading to pool GM and CSF together here since ihMTR is usually low in GM (but may become high using dedicated ihMT implementation using low duty cycle RF irradiation and no short-T1D filtering) but still give rise to detectable signal, whereas it is null or artefactual in CSF. Please rephrase.

 Head Orientation: “There was no significant interaction between angle and ROI (F = 1.47, p = 0.15) indicating effects were relatively homogeneous over the tracts.” This observation is surprising since each tract has a single orientation with respect to B0 and the orientation may differ between the right and left hemisphere; hence one expects different effects. Please discuss this point further. In addition, authors should provide more insight into the distribution of the head orientation within the tested population, e.g. by indicating the mean and standard deviation.

  Discussion:

 “Although blood is known to exhibit an MT effect, due to a high concentration of protein47, to our knowledge this is the first evidence that the effect in blood is significantly asymmetric. Although we do not discount the possibility that this is caused by inflow of unsaturated blood, it is difficult to see how this could produce a differential effect between the positive and negative offset frequencies, … “. I would be cautious about this interpretation since direct saturation and inflow effects may bias MT asymmetry. Again direct saturation effects are a potential concern with the presented sequence implementation (see general comment above), and may explain the observed signal. We encourage the author to perform in-vitro experiments on blood sample (if possible) to support this statement or to rephrase it.

 “there are no apparent drawbacks to switching from ihMTR to inverse ihMTR for future studies.” I agree there is no apparent drawback in terms of scan time, as describe here, but the advantages of inverse ihMTR in terms of robustness to B1 still need to be further supported by experiment. Consider rephrasing.

 “ihMTR have good, but not excellent repeatability…“. Authors have to describe repeatability in a more quantitative way.

 “This variation can at least in part be attributed to the orientation of the head with the main magnetic field. “ and “Despite these shortcomings, we showed a strong effect of angle on inverse ihMTR”. We invite the author to discuss more other sources of bias (residual B1 dependency, residual motion..) and to discuss the effect of the head orientation in a more quantitative way (e.g. compare the intra- vs -inter-session bias), rather than stating that the effect is “strong”.

 All figures from figure 3 appear too late in the pdf file; also figures 4 to 8 are all placed together.

Is the rationale for developing the new method (or application) clearly explained?

Yes

Is the description of the method technically sound?

Partly

Are the conclusions about the method and its performance adequately supported by the findings presented in the article?

Partly

If any results are presented, are all the source data underlying the results available to ensure full reproducibility?

Yes

Are sufficient details provided to allow replication of the method development and its use by others?

Partly

Reviewer Expertise:

OMG is an expert of MR physics and inhomogeneous manetization transfer (ihMT) MRI. LS is a expert of MR physics and ultra short echo time MRI.

We confirm that we have read this submission and believe that we have an appropriate level of expertise to confirm that it is of an acceptable scientific standard, however we have significant reservations, as outlined above.

## References

[1] : Magnetization transfer from inhomogeneously broadened lines (ihMT): Improved imaging strategy for spinal cord applications. *Magn Reson Med*.77(2) : 10.1002/mrm.26134 581-591 10.1002/mrm.26134 26959278

[2] : 472 - Anisotropy of inhomogeneous Magnetization Transfer (ihMT) in White Matter.2017;ISMRM 25th Annual Meeting:

[3] : Orientation dependence of magnetization transfer parameters in human white matter. *Neuroimage*.2015;114: 10.1016/j.neuroimage.2015.03.068 136-46 10.1016/j.neuroimage.2015.03.068 25862261

[4] : Whole brain inhomogeneous magnetization transfer (ihMT) imaging: Sensitivity enhancement within a steady-state gradient echo sequence. *Magn Reson Med*.79(5) : 10.1002/mrm.26907 2607-2619 10.1002/mrm.26907 28940355

# Response

**Published:** 2020-08-13

**Authors:** Tobias C. Wood

**Affiliations:** King's College London, UK

We thank the reviewers for their time and insight. There were in total five reviewers, with many helpful suggestions, and hence there have been many edits to the paper. Responses to this particular review follow below.

*1. About the systematic error observed in CSF*

We thank the reviewers for their suggestion that the negative effect in CSF may be caused by differential direct saturation effects from the single- and dual-sided irradiation. We agree that this is the most likely cause and the discussion has been amended as such. We agree that the suggested phantom experiment could clarify the cause, however due to the ongoing situation with Covid-19 we have not been able to acquire such data. As discussed further below, the issue can likely be mitigated in future studies by using a different RF pulse envelope, e.g. Hann or Gaussian.

*2. On the head orientation study*

We have reduced the emphasis on the head orientation study. The only message we intended to convey was that attention should be paid when positioning the subject. The word “strong” has been replaced with “highly statistically significant”, as this is a more accurate description of our result – which was a small effect but with a very small p-value. The abstract, introduction and discussion have been rephrased accordingly.

*3. Comparison with existing literature*

The introduction and discussion have been expanded to better explain the differences between a standard Cartesian gradient-echo sequence and the 3D radial sequence presented here. In response to another reviewer a table has been added with mean ihMTR and inverse ihMTR values from the ROIs which will make comparison with other papers easier. The achieved ICC values have been set in context against a Cartesian ihMT study that also used ICC scores.

We will address simulations of the RUFIS sequence in future work. Efficiently accounting for the combined action of the large number of readout pulses is an essential and general issue for Magnetization Prepared RUFIS and not specific to ihMT, and hence we consider it beyond the scope of this specific work. As the reviewers note it is essential that the readout pulses must have low RF power, and this was a key step in achieving good ihMT contrast (discussed further below).

*Specific comments:*

*Introduction: The introductory part of the angular dependency of ihMT should be expanded*. 

Thank you for the suggested references which have been added to the introduction

*Method/MR sequence: Author should provide more details about the “transient suppression” method that they used (fig. 1C), and provide argument or simple simulations so as to determine why “48 dummy segments” are sufficient to generate a steady-state.*

The description of the transient suppression has been expanded in the methods section and a justification for the 48 dummy segments provided (that 48 segments corresponds to 3.3 seconds of acquisition time which is much longer than T1 in brain parenchyma).

*Method/Imaging Study: Did the author consider adding the same spoiler gradient for the ihMT prep? (see general comment above)*

Originally the sequence included the same spoiler gradient for the MT-prep. However, a short spoiler generated considerable acoustic noise while a long (quiet) spoiler added appreciable dead-time to the sequence. We hence investigated the possibility of removing the explicit spoiler gradient and relying on the gradient ramp at the start of the acquisition segment, which at 5 ms long, has a sizeable gradient area. In our experience, insufficient spoiling manifests as wave/zipper type artefacts visible across the whole image, and as we did not observe such artefacts in our images, we concluded that spoiling from the ramp was adequate.

*Method/analysis: “As a proxy for how each subject’s head was aligned with the main magnetic field, we calculated the angle between the Z-direction (head-foot axis) of the MT scan space and the MNI atlas.“ This is an interesting approximation. However, in my opinion the main issue is that it misses the assessment of individual tracts orientation, which is necessary to address the angular dependency of ihMT.*

We agree that in order to fully model the angular dependency of ihMT the individual tract orientation should be known. It was not possible within the time budget of the protocol to acquire the diffusion data that would have provided this information. However, the fact that we found a significant interaction of head orientation implies that there is a measurable impact of head orientation on ihMT. As noted above, we have revised the wording to more correctly state that the effect was small but significant, and we hope that this is acceptable. 

*Method/Analysis related to the head orientation study: Some part of this section should appear in introduction as they are not specific to the method:*

The specified sentence has been moved to the introduction.

*Unfortunately, the B1 sensitivity of the high flip angle reference inverse ihMT ratio (ihMTRinv) is still under investigation and may not be as robust as initially thought. In addition, the sequence may have different sensitivity to B1 depending on the sequence parameters (see Mchinda et al. MRM 20184). In the presented study the ihMTRinv images from fig 2 show a strong hyperintense area located in the middle of the brain which looks somewhat colocalized with typical B1+ patterns observed @ 3T, as for the corresponding ihMTR images. This raises concern regarding the choice of ihMTRinv instead of ihMTR for the analysis. In addition, the residual dependency of ihMTRinv to B1 + shall be further discussed.*

The use of the inverse ihMTR metric was a best-efforts attempt to use the most recent recommended methods from the literature. Similar inverse metrics have been used previously in CEST to correct in particular for T1 relaxation, we have added a reference to a relevant paper in the methods section. Our results (higher ICC values) indicate that the inverse ihMTR is certainly no worse, and likely more robust than the ihMTR. Figure 2 has been revised to show axial and coronal sections, we hope that this makes it clear that the hyperintense area in the middle of what was the 3 ^rd^ slice is an ascending WM tract. We have reviewed Mchinda et al 2018 and can find no discussion of the inverse ihMTR within. A full characterisation of the B1+ variation would have required acquiring B1+ maps which was beyond the scope of this study. This has been listed as a limitation of the study in the discussion.

*Discussion: …Of interest, the MT experiments run in this study are not comparable to usual MT since relatively high frequency offsets and low duty cycle were used here…*

We thank the reviewers for pointing this out, it was also raised in another review. The discussion has been amended to make it clear that the offset frequency is optimal for ihMTR and not MTR.

*Discussion: Previous literature has shown that ihMT depends on many sequence parameters. This should be contextualized here. Also it is hard to compare with previous literature without providing the rms B1 calculated over the sequence repetition time.*

We have now contextualised our parameters with reference to Mchinda et al. in the discussion. The rms B1 over the preparation module has been added (discussed further below).

*Minor points: Abstract: Comparable should be removed.*

Removed as suggested.

*Introduction: Please specify “central nervous system”.*

Added as suggested.

*p3: reference to citation 11 about ihMT is likely inappropriate.*

The citation was in the incorrect place. Thank you for spotting

.

*Methods: Please use “mitigate” instead of “avoid”.*

Changed as suggested.

*MR sequence: “... increased ihMT effect in myelin ” Please use WM instead of myelin, this is a shortcut toward considering that ihMT is a purely myelin-specific effect.*

We are unsure what the specific issue is here. We have amended the sentence to read “increased sensitivity to myelin”, and to reference the paper “Validating the sensitivity of inhomogeneous magnetization transfer (ihMT) MRI to myelin with fluorescence microscopy”, Duhamel et al 2019

*Imaging study: typo: plural “6 females, 6 males”.*

In this context female and male are not nouns but adjectives referring to the implied plural noun “subjects”. We have amended the sentence to make this explicit.

*Imaging study, first paragraph: we suggest to make explicit here the reason why the ihMT scans were repeated twice (repeatability) and repeated again in another session (head orientation study).*

This is an incorrect interpretation. All four measurements (two scans x two sessions) were used for the repeatability study, and then also included in the head orientation study.

*Imaging study: “We used a 2°, 24μs (low B1 amplitude) hard pulse for excitation to minimize any saturation of the bound pool from the excitation pulses”: the sentence sounds misleading since shorter RF pulses lead to stronger B1RMS for identical flip angle, please rephrase.*

The 24 μs pulse was lengthened from the manufacturer default of 8 μs, in order to reduce the B1 amplitude and consequent on-resonance saturation of the MT pool. This has been made explicit in the text.

*Imaging study: was the choice of Fermi pulse motivated by any specific criteria? Since there are free parameters in the Fermi window function, the authors should provide more information on the pulse shape, such as bandwidth and power integral (or B1peak and B1rms is preferred).*

Fermi pulses are used by our scanner manufacturer in some sequences and there was no specific criteria beyond the desire to maximise the achievable B1rms within a given pulse-width. The B1rms of the pulse (8.75 uT) is stated in the methods section (Imaging Study). Given the single/dual-sided irradiation issue identified by the reviewers above, switching to an envelope with lower sidebands, e.g. Hann or Gauss, is preferable for future studies, and this has been noted in the discussion.

*Imaging study: please provide the root-mean-square B1 of MT pulses calculated over the segment TR (ihMT prep + readout) to ease comparison of presented results with existing literature.*

Previous literature has used a wide variety of power metrics. In particular Mchinda et al used the rms B1 over the preparation module, not the segment TR. As we have used Mchinda et al as a reference point for several other features of our sequence, we have added the relevant figure (6.2uT) to the methods section.

*Imaging study: we suggest the author describe first that ihMT rely on comparing single and dual frequency MT experiment, before describing the five volumes to be acquired. Currently the ihMT method description may be difficult to follow for non-expert ihMT users.*

The suggested sentence has been added to the methods section.

*Analysis: reference to citation 36 about the QUIT toolbox is likely inappropriate. Please check all references.*

We wrote a new tool specifically for calculating MT ratios and added it to QUIT. This has been clarified in the text. The documentation for this tool is available here: https://quit.readthedocs.io/en/latest/Docs/MT.html#qi-mtr

*Analysis: “Previous work has shown that the inhomogenous MT effect partially depends on the orientation of myelin with the main magnetic field”. typo: “inhomogeneous”.*

Thank you for spotting the typo, in the course of responding to other comments the word has been removed.

*Results:*

*Images: No acoustic noise measurement was reported for the T1w-RUFIS acquisition. p6 typo error: “The CoV in WM was below 10% in WM”. WM is repeated twice.*

The T1w acquisition used the same readout module with the same acoustic characteristics as the MT module. The typo has been corrected.

*“the very small absolute values of ihMTR in both GM and CSF, ...”..It is misleading to pool GM and CSF together here since ihMTR is usually low in GM (but may become high using dedicated ihMT implementation using low duty cycle RF irradiation and no short-T1D filtering) but still give rise to detectable signal, whereas it is null or artefactual in CSF. Please rephrase.*

The mention of CSF has been removed.

*Head Orientation: “There was no significant interaction between angle and ROI (F = 1.47, p = 0.15) indicating effects were relatively homogeneous over the tracts.” This observation is surprising since each tract has a single orientation with respect to B0 and the orientation may differ between the right and left hemisphere; hence one expects different effects. Please discuss this point further. In addition, authors should provide more insight into the distribution of the head orientation within the tested population, e.g. by indicating the mean and standard deviation.*

“Tract” has been replaced by “ROI”, this is subtle but important point as ROIs may contain multiple tracts. We have added a note that we did not separate ROIs by hemisphere as a limitation in the discussion. The median head angle was stated in the results section, the lower and upper quartiles have been added.

*Discussion: I would be cautious about this interpretation since direct saturation and inflow effects may bias MT asymmetry. Again direct saturation effects are a potential concern with the presented sequence implementation (see general comment above), and may explain the observed signal. We encourage the author to perform in-vitro experiments on blood sample (if possible) to support this statement or to rephrase it.*

We have found additional references detailing the MT effect in blood and describing an asymmetric profile. These have been added to the discussion and we no longer claim to be the first to observe this effect.

*“there are no apparent drawbacks to switching from ihMTR to inverse ihMTR for future studies.”* *I agree there is no apparent drawback in terms of scan time, as describe here, but the advantages of inverse ihMTR in terms of robustness to B1 still need to be further supported by experiment. Consider rephrasing.*

This sentence has been removed while responding to other comments.

*“ihMTR have good, but not excellent repeatability...“. Authors have to describe repeatability in a more quantitative way.*

“Good” and “Excellent” refer to a standardised ICC classification score (ICC value 0.75-0.9 good, > 0.9 excellent). This has been clarified and the relevant citation added.

*We invite the author to discuss more other sources of bias (residual B1 dependency, residual motion..) and to discuss the effect of the head orientation in a more quantitative way (e.g. compare the intra- vs -inter-session bias), rather than stating that the effect is “strong”.*

As noted above, we have revised our wording to “small but highly significant”. Strong was used in reference to the small p-value, we hope the new wording is clearer.

*All figures from figure 3 appear too late in the pdf file; also figures 4 to 8 are all placed together.*

Final figure placement was determined by the journal and beyond the control of the authors.

# Peer review: Reviewer response for version 1

**DOI:** 10.21956/wellcomeopenres.17381.r38493

**Published:** 2020-05-14

**Authors:** Richard Dortch

**Affiliations:** 1 Division of Neuroimaging Research, Barrow Neurological Institute, Phoenix, AZ, USA

This well-written manuscript seeks to develop and evaluate a silent myelin-specific MRI sequence for applications in infants and the elderly, where loud imaging sequences can be problematic. Recent work has demonstrated that so-called inhomogeneous MT (ihMT), which arises primarily from dipolar order effects in myelin lipids, may be a more specific assay of myelin content than other MRI measures (e.g., T _2_ relaxation, diffusion, conventional magnetization transfer). As a result, there is significant interest in developing clinically feasible ihMT sequences for applications in neurodegenerative diseases, development, and aging. Overall, the study was well designed (e.g., strong repeatability and ROI analyses) and the results were compelling. However, there are several minor-to-moderate flaws, particularly in the motivation (e.g., the need for silent ihMT sequences) and methods (e.g., the influence of head orientation on ihMT), that slightly reduced my enthusiasm and lead me to recommend a minor revision.

1. The case made for silent MT sequences is not particularly compelling. The authors mention that these are “among the loudest” sequences because they use fast gradient-echo readouts to obtain whole-brain data in clinically feasible scan times. However, these sequences are usually SAR-limited with fairly reasonable TRs (typically between 25-50 ms) that are acquired at lower resolutions to ensure adequate SNR. Together, this results in a sequence with reduced acoustic noise compared to most rapid, high-resolution gradient echo sequences as well as other quantitative approaches that use EPI (e.g., diffusion). (moderate)
2. Furthermore, the benefits of using a silent myelin sequence may not outweigh the drawbacks. For example, the proposed method requires very low flip angles (2 degrees), which results in a significant SNR penalty relative to standard ihMT sequences. In addition, the RUFIS readout results in a small increase in scan time. Given than SNR is already relatively low for ihMT indices, the proposed method may be suboptimal in many clinical scenarios. (moderate)
3. The study was not designed to specifically measure the effect of head orientation on ihMT. Subjects were scanned four times (across two sessions), but head orientation was not directly controlled or measured across these scans. Instead a mixed effects model was used and head orientation was inferred from the images (rather than the orientation of individual tracts being measured using DTI for example). Furthermore, the confounding influences of T _1_ and B _1_ were not measured. The authors attempt to overcome this by using inverse ihMT, which is less sensitive to these confounding influences. However, the inverse ihMT maps in Figures 2 and 4 show some shading artifacts that may be related to uncorrected B _1 _variations. (moderate)
4. Results from the silent sequence were not compared to conventional (i.e., “loud”) sequences, both in terms of acoustic noise and MT parameters. (moderate)
5. Figure 1: the “stair-stepping” of gradient amplitudes is difficult to see in panel A. (minor)
6. The same offset (7 kHZ) and RMS power was for the saturation pulses in ihMT and MTR acquisitions. For MTR, saturation pulses are typically applied at 1-2 kHZ off-resonance relative to water to maximize MT contrast; therefore, the authors are comparing a sequence that may be optimized for ihMT to a suboptimal MTR sequence. (minor)
7. It was not clear why spoiling was required for the T _1_-weighted scan but not the MT scans. (minor)

Is the rationale for developing the new method (or application) clearly explained?

Partly

Is the description of the method technically sound?

Yes

Are the conclusions about the method and its performance adequately supported by the findings presented in the article?

Partly

If any results are presented, are all the source data underlying the results available to ensure full reproducibility?

Yes

Are sufficient details provided to allow replication of the method development and its use by others?

Yes

Reviewer Expertise:

Imaging science, quantitative MRI, myelin imaging.

I confirm that I have read this submission and believe that I have an appropriate level of expertise to confirm that it is of an acceptable scientific standard, however I have significant reservations, as outlined above.

# Response

**Published:** 2020-08-13

**Authors:** Tobias C. Wood

**Affiliations:** King's College London, UK

We thank the reviewer for their time and insight. There were in total five reviewers, with many helpful suggestions, and hence there have been many edits to the paper. Responses to this particular review follow below.

1. We concede that the acoustic noise from any scan will depend on the precise sequence settings. However, we note that recent ihMT work has used both an MP-RAGE style acquisition, with an imaging TR of 4.3ms and also SSFP with a TR of only 5ms. The introduction has been amended to explicitly reference these papers.

2. We agree that radial sequences are SNR constrained relative to cartesian sequences, this has now been explicitly stated in the discussion. Although the 3D radial readout does imply a time penalty relative to cartesian, we note that our overall scan time is competitive with recent cartesian ihMT papers. This has been added to the discussion.

3. We agree that it would have been preferable to acquire explicit T1 & B1 maps for comparison, but total protocol time prevented that in this study. In our opinion the ihMTRinv maps display more even contrast than the ihMTR maps, we hope that the revised figures with axial and coronal sections make this clearer. 

4. We did not have a conventional cartesian ihMT implementation available when this study was conducted. However, as there are multiple such implementations in the literature, it is possible to broadly compare image quality and achieved ihMTR values. We have added a table of ihMTR values to make this comparison easier. We concede that it is not possible to compare acoustic noise levels, because it is not standard in the MR literature to record and report the acoustic noise of a sequence. In previous work (reference 22) we did directly compare noise levels between a radial ZTE and cartesian implementation of Variable Flip-Angle T1 mapping, which in our opinion would be similar to the noise levels in this work and found a 30 dB reduction in noise level.

5. Figure 1 has been updated with a reduced number of spokes to emphasise the stepped gradients. We hope this is clearer.

6. We thank you for pointing out that the frequency offset is not ideal for generating single-sided MT contrast. With hindsight, this is obvious. The discussion has been amended to reflect this.

7. Because the MT pulses are applied off-resonance they should not significantly interact with the free water pool and hence generate a minimal amount of transverse magnetization that would require spoiling – in addition, the initial gradient ramp of the acquisition segment does provide some spoiling in a quasi-random direction. In contrast the T1-preparation pulse does excite a large amount of transverse magnetization (because it is applied on-resonance), which must be sufficiently spoiled before the start of the acquisition segment. We have added additional sentences to the methods to make this clearer.
